# Supplementary material for: MetaRibo-Seq measures translation in microbiomes
Source: Nat Commun. 2020 Jun 29;11:3268. doi: 10.1038/s41467-020-17081-z (PMC7324362; doi:10.1038/s41467-020-17081-z)
Supplement: Supplementary file 10 — Supplementary Data 7 [file 41467_2020_17081_MOESM10_ESM.zip › File2/Confidence_VeryHigh_Taxonomy/207272_out.krona.html]

Javascript must be enabled to view this page.

members
magnitude
magnitudeUnassigned
count
unassigned
taxon
rank

207272\_out

31

31
2
superkingdom

1239
phylum
31

186801
class
31

31
186802
order

29
family
186803

genus
841
29

360807
species
29

SRS015486\_contig\_number\_contig-100\_2552.2553SRS015663\_contig\_number\_4733SRS015890\_contig\_number\_16686SRS018817\_contig\_number\_contig-100\_2452.116603SRS018984\_contig\_number\_22934SRS019445\_contig\_number\_5776SRS019910\_contig\_number\_12160SRS022524\_contig\_number\_contig-100\_16111.47818SRS022609\_contig\_number\_28221SRS046502\_contig\_number\_contig-100\_12510.12511SRS052697\_contig\_number\_30862SRS054905\_contig\_number\_8830SRS063040\_contig\_number\_43900SRS077194\_contig\_number\_14716SRS077552\_contig\_number\_20254SRS078419\_contig\_number\_37389SRS097889\_contig\_number\_28841SRS098881\_contig\_number\_26404SRS1041032\_contig\_number\_contig-100\_1260.81742SRS1041038\_contig\_number\_3259SRS1041130\_contig\_number\_contig-100\_6.46190SRS1055069\_contig\_number\_contig-100\_1758.64898SRS140513\_contig\_number\_contig-100\_1151.39716SRS143417\_contig\_number\_24428SRS143780\_contig\_number\_26243SRS143991\_contig\_number\_contig-100\_25692.77016SRS147346\_contig\_number\_62337SRS148424\_contig\_number\_contig-100\_36492.36492SRS893342\_contig\_number\_contig-100\_12.70473

186806
family
1

1
1730
genus

1897004
species
1

SRS020328\_contig\_number\_3566

1
family
31979

genus
1649459
1

1

SRS146888\_contig\_number\_14589
154046
species
